# Measuring linguistics of the *wokototen* chart made inductively by deciphering *kunten* materials

**DOI:** 10.12688/f1000research.131244.1

**Published:** 2023-05-16

**Authors:** Tomoaki Tsutsumi, Koji Tajima, Teiji Kosukegawa, Tomokazu Takada

**Affiliations:** 1 Faculty of Humanities and Social Sciences, University of Tsukuba, Tsukuba, Ibaraki, 305-8577, Japan; 2 Department of Electrical and Computer Engineering, National Institude of Technology(KOSEN), Gifu College, Motosu, Gifu, 501-0495, Japan; 3 School of Humanities, University of Toyama, Toyama, Toyama, 930-8555, Japan; 4 National Institute for Japanese Language and Linguistics, Tachikawa, Tokyo, 190-0014, Japan

**Keywords:** Kunten, Reading Classical Chinese in Japanese, Text Mining, Digital Humanities

## Abstract

In this paper, we focus on *wokototen* markings, which are a system of *kunten* annotations used to facilitate the reading of classical Chinese documents by Japanese readers. Using digitized data, we performed basic measurements of wokototen by using a chart that summarizes the *wokototen* markings of actual *kunten* materials described by Hiroshi Tsukishima, and we quantitatively clarified their characteristics. *Kunten* materials are classical Chinese books with annotations, called *kunten*, on the Chinese text. The *wokototen* is a type of *kunten*. In ancient East Asian countries, *kunten* systems were developed as a way of directly annotating Chinese documents so that they could be read and understood by non-native readers. For this reason, *kunten* materials and *kunten* are treated as historical sources for linguistic and historical research. The shape and position of a *wokototen* marking determines what kind of reading it indicates. The results of our basic survey quantitatively show that almost all the *wokototen* charts in actual *kunten* materials contain particles represented by “te”, “ni”, and “wo”, the most common shapes of *wokototen* are dots and shapes that can be written with a single stroke, such as ｜, ─, and ＼, and that the most common places to find these markings are to the right of characters in the horizontal direction and below characters in the vertical direction.

## Introduction

*Kunten* is a Japanese system of text markings used to clarify the syntax and meaning of Chinese texts for Japanese readers. Previous research ^1^^–^^3^ investigated how these marks can be handled on a computer. In this paper, we focus on a *kunten* system called *wokototen* that is used to annotate classical Chinese texts ( *kanbun*), and we perform basic measurements by digitizing *wokototen* charts ( *wokototen-zu*) that inductively summarize the markings added to actual documents.

The texts we are studying consist of classical Chinese texts annotated with *kunten* markings. These were once widely used, around from the Heian period to the Edo period in Japan, to promulgate social, cultural, and academic ideas in East Asian countries, where *kunten* systems were developed as a way of directly annotating these documents so that they could be read and understood by non-native readers.

*Wokototen* is one such system that was developed in Japan. It consists of marks placed inside or around the Chinese characters to indicate features such as grammatical particles, auxiliary verbs, and the readings of kanji characters. There are various flavors of *wokototen* associated with different schools and different annotators, and *wokototen* charts are used as means of inductively compiling the annotation marks used by different schools of *wokototen* or in different *kunten* documents.

Documents annotated in this way have existed from the Heian period to the present day, and *wokototen* is mostly found in Chinese classics, Buddhist scriptures, and Japanese books of the Heian and Kamakura periods. Since the materials that are the subject of our research contain complex information, this information must be preserved intact when they are digitized for analysis purposes. We therefore digitized the data by using a dedicated structured description method. For this paper, particularly with regard to this digitized data, we performed basic measurements of *wokototen* by using a chart that summarizes the *wokototen* markings of actual *kunten* materials described by Hiroshi Tsukishima, ^4^ and we quantitatively clarified their characteristics.

## Target of research

### Overview of *wokototen*

*Wokototen* markings are often found in *kunten* documents from the Heian and Kamakura periods, where they are used to indicate features such as grammatical particles, auxiliary verbs, and conjugated endings by means of variously shaped symbols such as dots (・), lines ( │ ), and hooks (└ ). These symbols can be placed at the four corners, inside, or around the strokes of a Chinese character, and their readings differ depending on their position and shape. For example, a dot to the upper right of a *kanji* has a different meaning than a line in the same position, or a dot to the lower right of the character. To uniquely identify the meaning of a *wokototen* markings, we need to know both its position and its shape.

There are various types of *wokototen* associated with different eras and different schools. For example, in one school, a dot at the upper right corner of a Chinese character is read as *wo*, but in another school, it is read as *koto.*

### *Wokototen* charts and the study of *kunten*

The decipherment and classification of *wokototen* has been the subject of several previous studies by researchers including Yoshinori Yoshizawa, ^5^^–^^7^ Norio Nakata, ^8^ Heiji Otsubo, ^9^ Hiroshi Tsukishima, ^4^^,^^10^ Yoshinori Kobayashi, ^11^^,^^12^ and Masaji Kasuga. ^13^ In particular, Norio Nakata ^8^ compiled a set of 26 *wokototen* charts as materials for deciphering *kunten* materials.

Even today, researchers specializing in *wokototen* are working to decipher *kunten* materials and study their historical background. Their general research method involves visually deciphering the *wokototen* and *kana* annotations in *kunten* materials to create Japanese transcriptions from which the contents of the materials can be understood. As a by-product of this process, a *wokototen* chart is generated as a key to the markings used in the source material. A *wokototen* chart not only summarizes the *wokototen* markings in the actual *kunten* materials, but also includes information on how the researchers understood these materials.

**Figure 1.**
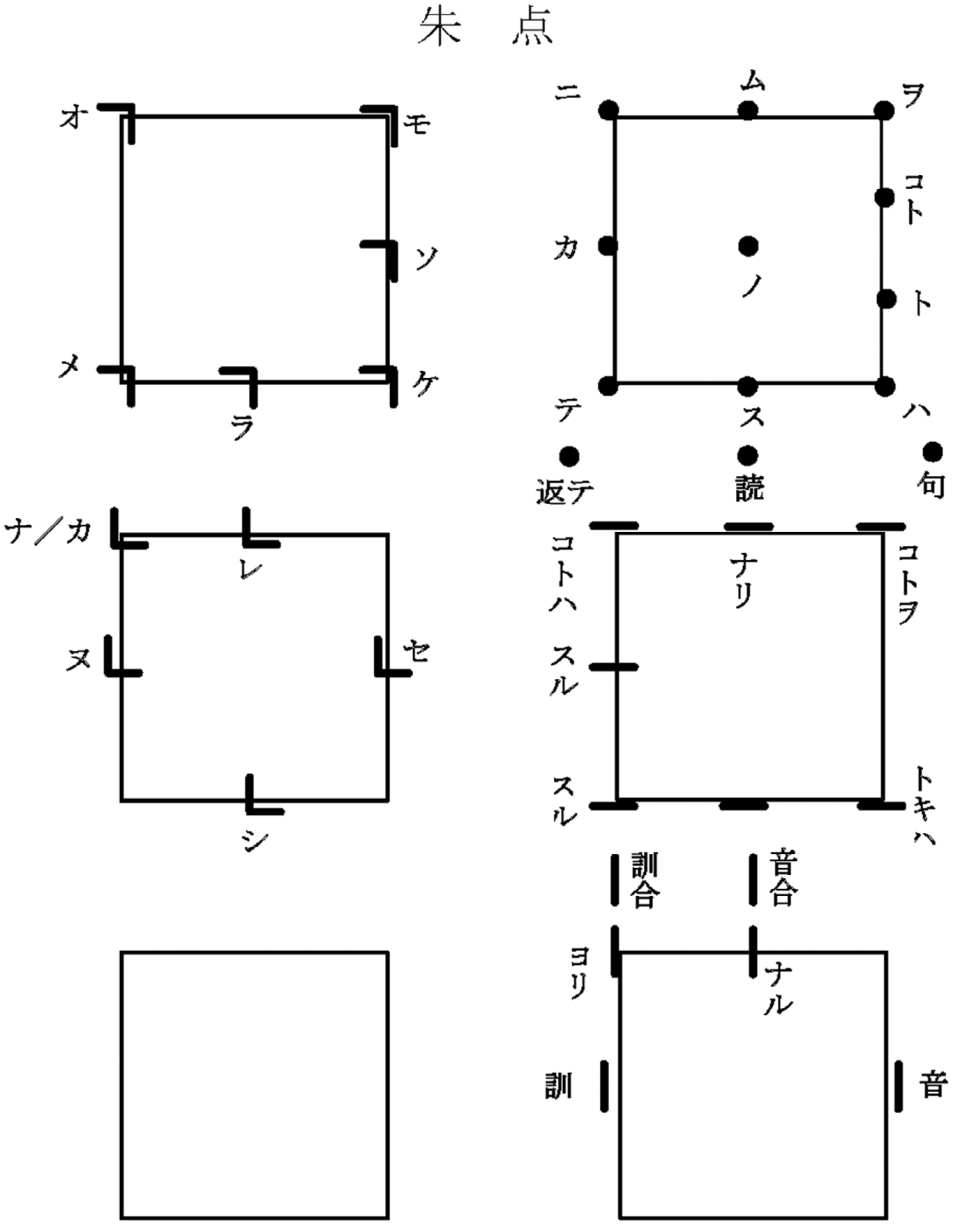
Example of a *wokototen* chart for *Kobunshōsho* (古文尚書), an important cultural property held at the Toyo Bunko Museum in Tokyo made by Teiji Kosukegawa.

As shown in Figure 1, a *wokototen* chart indicates the reading of each marking according to its shape and position in a square frame (called a *tsubo*) corresponding to the location of a Chinese character. A *wokototen* chart typically consists of multiple tsubo, with each *tsubo* containing multiple *wokototen.* A collection of these *wokototen* charts is called a *tenzushū* (点図集).

*Wokototen* charts can be classified into two types according to the process by which they were created. One is a comprehensive chart, which is a collection of the *wokototen* markings used by each school. The other is an inductive chart, which is a collection of the *wokototen* markings used in a particular body of *kunten* material. The measurements in this study were made using the latter type of chart. For this paper, we performed basic measurements on 199 types of point charts contained in actual *kunten* materials as summarized by Hiroshi Tsukishima. ^4^ By way of comparison, we also present the results of measurements made using the former type of point chart as described by Tomoaki Tsutsumi. ^1^

## Methods

### Digitization of *wokototen* chart data

The data used in this study was based on *wokototen* charts data created from *kunten* materials created by Hiroshi Tsukishima, which were registered as of June 2022 in the *Wokototen* charts Database provided by the National Institute for Japanese Language and Linguistics (NINJAL). Further information on the database can be found on the NINJAL website and in reference [ 3].

As mentioned above, *wokototen* markings are used to annotate Chinese characters. The meaning of each marking is determined by its position, shape, and reading. In the data of NINJAL, the 'reading' of the *wokototen* has been entered in Japanese text. The ‘shape’ of the *wokototen* has been entered by replacing it with the similar character in Unicode. The 124 characters substituted in this database is shown in Table 1. The position of a *wokototen* is represented using a 7×7 square grid of cells with its origin at the center of a *tsubo* and the upper left and lower right corners at the coordinates (−3, −3) and (3, 3). Since *wokototen* markings are sometimes slightly separate from the kanji character, the center 5×5 square corresponds to the area occupied by the character, and coordinates in this region correspond to markings that overlap with the character. The outermost cells are used for positions that are separate from the kanji character. This digitization method of the readings, positions and shapes of the *wokototen* are described in detail in reference [ 1].

**Table 1.** List of shapes used for digitisation.

Shape										
・	┼	七	/	丁	⠑	ア	ᓓ	カ	ހ	┼又は┤
｜	：	レ	╰	〝	ム	4	॥	マ	い	ဂ
—	〢	ナ	—↑	ク	リ	丿	⨽	∧	下	凵
＼	〇	ノ	ᄓ	⠔	┤	つ	…	٦	ᨀ	⍭
└	乙	◠	⧷	ﾌ	∴	∠	y	⎞	山	N
／	├	ル	イ	ス	コ	⍀	匚	◠↓	⦧	亽
┐	＝	┴	×	ヒ	＞	┌	上	৲	-	冖↓
フ	┬	⧹	⊂	—↓	へ	ヌ	〃	C	丶	ち
人	⊃	∨	⌵	┘	口	∪	⦣	寸	牛	ケ人よこ
‥	◡	ソ	<	⦦	╲	〵	⁐	ᓗ↑	ᚇ	く
⌰	∅	⎱		ﬧ	匕	ヤ	う	⎵	∵	又ハ┤
ヘ	～	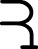								

This method of expressing the position of the Kunten in coordinates has also been used in Korean studies of *gugyeol,* ^14^^–^^18^ where a coordinate system of 5×5 squares is defined around each character. These methods of the Korean studies and the current one has the same concept but are not data compatible.

### Basic measurements for *wokototen* charts

In this study, we performed measurements on 199 types of point charts (contained in actual kunten materials as summarized by Hiroshi Tsukishima ^4^), relating to the reading, position, and shape of *wokototen.* The programme written in C# was used for the measurements. ^19^ Excel 365 and Visual Studio Code (version 1.67) were used to view and compare the data from the database with the reference [ 4] and to correct the data. VisualStudio Community (version 17) was used to create the programme.

This program reads comma-delimited data, one per line, on the *wokototen* to be measured. It is a simple program that outputs the count of “reading,” “position,” and “shape” of the *wokototen.* A line of read data should be produced in the following format.

Material Title,Tsubo No,Reading,Sharp,Position of X, Position of Y

*Ex.* 華厳経探玄記 一巻,1,レ,・,-3,-3

We created this form of data by comparing data taken from the *Wokototenzu* database with the previous research. ^4^ In some cases, there were multiple readings given for a single symbol in the *wokototen* chart. Examples are shown in the 'カ/ナ' in the top left-hand corner (-2, -2) of the fifth tsubo in Figure 1. In this case, we have divided the symbol into two separate totals. The one of *wokototen* of the shape ‘L’ reading ‘カ’ and the other of *wokototen* of the shape 'L' reading 'ナ. In the data created by adding these processes, the total number of target *wokototen* was 6411.

## Results

### Results for reading of *wokototen*

The readings of *wokototen*, such as “wo” and “koto,” were examined to determine how many were found in the target *wokototen* chart. As a result, there were 303 types of readings. Next, the number of these 303 types of readings on the *wokototen* chart were measured, and the top 10 types are shown in Table 2. This does not include 885 points for which no reading was noted.

**Table 2.** The 10 most common *wokototen* readings.

reading	Occurrences	reading	Occurrences
Te(テ)	203	Ha(ハ)	181
Wo(ヲ)	199	Su(ス)	166
Ni(ニ)	199	Koto (コト)	159
To(ト)	191	Ru(ル)	155
No(ノ)	190	Nari (ナリ)	149

### Results for shapes of *wokototen*

We examined the shapes of *wokototen* markings such as ・ and │ to determine how many of each there were in the target *wokototen* chart. As a result, we found 124 different shapes. We then counted the occurrences of each of these shapes in the *wokototen* chart, and the top 10 shapes are shown in Table 3. Since the *wokototen* charts used in this study were hand-drawn, there were several shapes that were partially similar but with slight differences. In such cases, we counted the shapes as being of a single type. For example, the shapes 人 and 入 were grouped together as 人 and counted as the same shape.

**Table 3.** The 10 most common *wokototen* markings.

Shape	Occurrences	Shape	Occurrences
・	2199	/	377
|	770	┐	264
—	525	フ	130
\	520	人	119
└	397	‥	107

Next, we examined the shapes used to indicate the three most frequent readings, which were “te”, “ni”, and “wo”. As shown in Table 4, we found that these readings were represented by seven shapes: ・, \, /, |, ─, ◡, and :. The ・ shape was the most common.

**Table 4.** Number of shapes used to represent the readings “te”, “ni”, and “wo”.

Shape	Te(テ)	Ni(ニ)	Wo(ヲ)
・	195	191	179
\	5	3	5
/	1	1	0
|	1	0	0
—	1	4	2
◡	0	0	12
:	0	0	1

### Result for positions of *wokototen*

**Figure 2.**
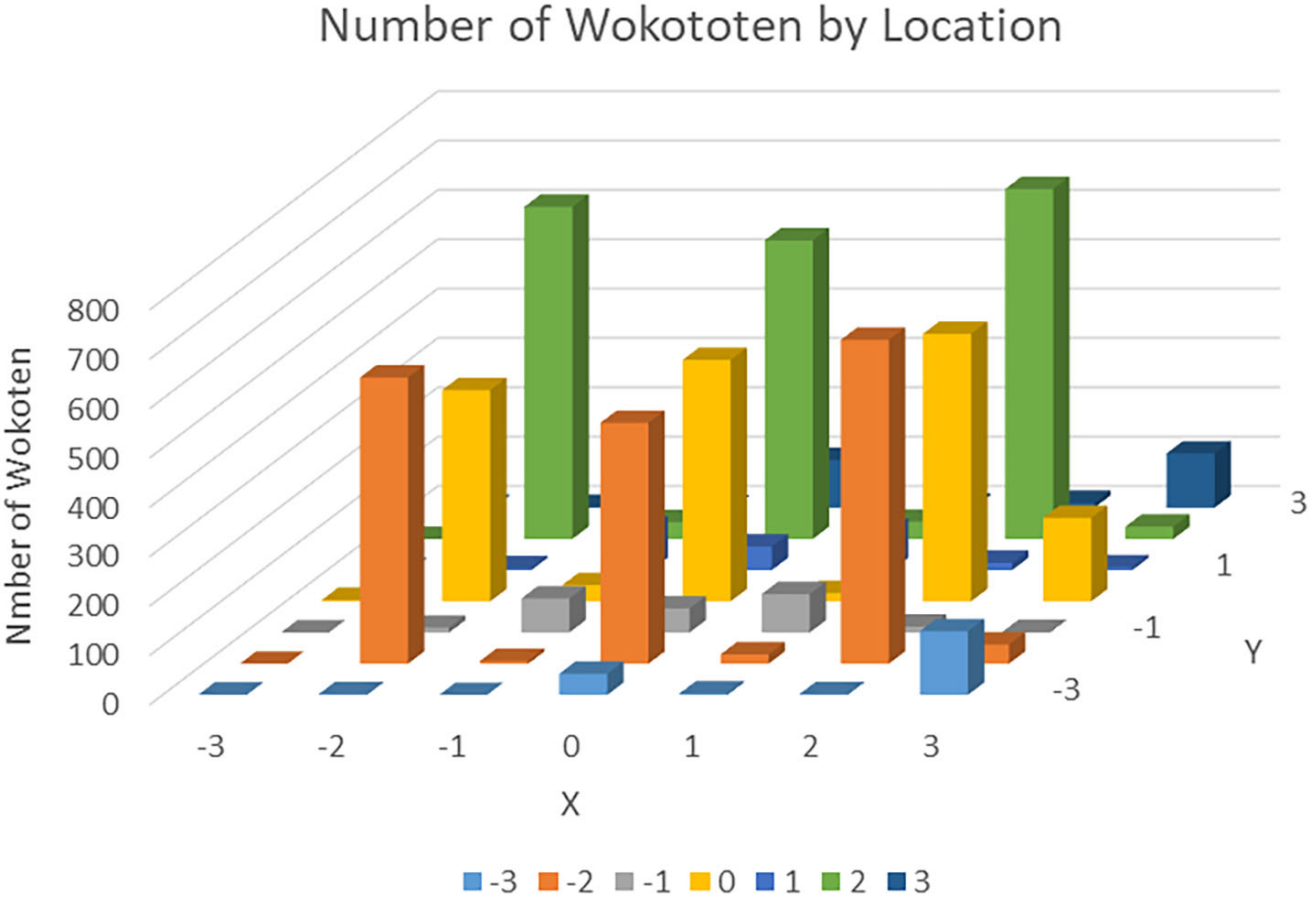
Number of *wokototen* by location (graph).

The results of measuring the locations of *wokototen* markings are shown in Table 5 and are depicted graphically in Figure 2. In Table 5, the area with the red background shows the position of the square cells representing the location of the kanji character.

**Table 5.** Number of *wokototen* by location.

Y/X	-3	-2	-1	0	1	2	3
-3	1	2	0	43	3	2	129
-2	0	580	6	488	19	656	39
-1	0	10	69	49	78	12	1
0	3	428	33	489	17	542	169
1	0	4	84	48	80	15	6
2	0	673	34	605	35	709	25
3	2	2	0	97	4	10	110

## Discussion

### Reading

As Table 2 shows, there are many particles and auxiliary verbs such as “te”, “ni”, “wo”, and “ha”. The most common reading was “te”, which had more appearances than the number of *wokototen* charts (199) because it sometimes appeared more than once in the same chart, and because there were only eight charts in which this reading was not mentioned. These were charts with an extremely small number of *wokototen* points. One example is Scroll 1 of the *Biography of Yang Xiong Zhuan* (漢書楊雄傳; yellow markings, 5th level), which has only one *wokototen* marking, Scroll 3 of the *Mohe Zhiguan* (魔訶止觀; green markings), which has eight, and Scroll 1 of *Huiguo Heshang Zhi Bei* (惠果和上之碑文; black markings), which has two. In cases where the markings appear in different colors, such as red/vermillion ( *shuten*), black ( *bokuten*), and white ( *hakuten*), the symbol “te” was marked in a different color. The same is true for “wo” and “ni”, which explains why “te”, “wo”, and “ni” in particular are found in almost all *wokototen* charts.

A comparison with the results of a survey ^1^ of 26 major *wokototen* charts compiled by Nakata ^8^ and Tsukishima ^4^ for each school shows that there are differences in the types of readings that occur frequently. The most common readings in the major *wokototen* charts are “su”, “naru”, “nari”, and “tari”, but these occur less frequently in *wokototen* charts based on *kunten* materials. Of these readings, the most common was “su” with 166 occurrences, followed by “nari” with 149. The other readings “tari” and “naru” were the 22nd and 24th most frequent, with 80 and 77 occurrences, respectively.

### Shapes

Table 3 shows that the overwhelming majority of *wokototen* markings are dots (・). There were six *wokototen* chart that did not contain dots. These were charts containing almost no *wokototen*, as in the *Biography of Yang Xiong Zhuan*, and charts where the \ shape was used for the first *tsubo* instead of a dot, as in Scroll 1 of *Tōdaiji Fujumonkō* (東大寺諷誦文稿) and Scroll 8 of *Myōhō Rengekyō* (妙法蓮華経; the *Lotus Sutra*).

Including the dot marking (・), which appeared most frequently, the *wokototen* shapes ・, |, ─, and \ that appeared more than 500 times are shapes that can be written with a single stroke. More complex shapes appeared less often, and the same trend was observed in the measurement results of the 26 main *wokototen* charts.

The results in Table 4 also show that readings with the highest number of appearances are often denoted by a single dot. Although other shapes are sometimes used, it is safe to say that these readings are almost always denoted by a dot in all schools. This is thought to be because particles that are important in reading Chinese texts as Japanese are often expressed in the simplest way, which means using a single dot, because of the large number of times they are added. In some cases, “wo” is represented using the ◡ shape, and these were the *wokototen* charts belonging to the fourth group in the classification according to Tsukishima. ^4^

### Position

From Table 5, it can be seen that *wokototen* are often drawn at the four corners and the center of a character. The most common location was the lower right corner (2, 2), where 709 *wokototen* were found. In the four corners of characters, where *wokototen* markings are often placed, markings were more commonly found in the lower corners in the vertical direction, and on the right side in the horizontal direction.

Next, we examined the *wokototen* markings placed around the outside of the characters. More markings were found on the right side than on the left side. For example, there were 129 markings in the upper right external position (3, −3), but only one in the upper left external position (−3, −3). The most common positions of *wokototen* markings around the outside of a character were the top, middle and bottom on the outer right side, with the largest number (169) appearing in the middle at coordinates (3, 0). These results show that *wokototen*, like *furigana*, tend to be written to the right side of each character.

The above trends suggested by our measurements differ from the results obtained from the 26 main *wokototen* charts, where there was no difference in the placement of markings between the left, right, upper and lower positions. In addition, measurements of the 26 main *wokototen* charts showed that few of these markings are placed outside the characters, while in the actual materials it can be seen that many *wokototen* markings are placed to the right of each character. These differences may represent a trend caused by the actual addition of markings to *kunten* materials.

## Conclusion

We have conducted basic measurements of *wokototen* markings as described by Hiroshi Tsukishima, ^4^ who compiled a chart summarizing the *wokototen* markings applied to actual *kunten* materials. As a result, we have quantitatively demonstrated that almost all *wokototen* charts in actual *kunten* materials contain particles represented by “te”, “ni”, and “wo”. Our results also show that the most common shape used for *wokototen* is ・, and that markings with shapes of greater complexity are used in fewer documents. We found that *wokototen* are most often placed at the bottom and right of characters, and that when they are placed outside the characters, they appear mostly to the right side of them, showing the same tendency as that of Japanese annotations such as *furigana.*

In the present analysis of the trends in the Wokotenzu charts, it has been possible to identify an overall trend. However, it cannot be determined whether all individual Kunten materials show a similar trend. Further research into the content of individual Kunten Material will be required in the future.

In the future, we plan to use the information revealed by these basic measurements to develop a system that will help researchers to guess and present the locations of *wokototen* markings when deciphering *kunten* materials, and to automatically generate written transcriptions to help readers understand these materials. Another future task will be to extract the characteristics of *wokototen* by using large amounts of data, which have been difficult to compare and study using conventional manual research methods.

## Data Availability

The data for this study is owned by the National Institute for Japanese Language and Linguistics. The data of *wokototen* charts were used this study and can be obtained and search here: https://cid.ninjal.ac.jp/wokototendb/. The data is available in accordance with the policy of the National Institute for Japanese Language and Linguistics. The policy is here: https://www.ninjal.ac.jp/english/utility/policy/. Analysis code available from: https://github.com/TTMTMAK/CountProg_for_Wokoto/releases. https://github.com/TTMTMAK/CountProg_for_Wokoto/tree/master. Archived analysis code at time of publication: https://doi.org/10.5281/zenodo.7801472. ^19^ License: Apache License, Version 2.0
