# Supplementary material for: Measuring linguistics of the wokototen chart made inductively by deciphering kunten materials
Source: F1000Res. 2023 May 16;12:506. [Version 1] doi: 10.12688/f1000research.131244.1 (PMC10521107; doi:10.12688/f1000research.131244.1)
Supplement: Peer review history

# Reviewer report: Reviewer response for version 1

**DOI:** 10.5256/f1000research.144061.r186342

**Published:** 2023-08-21

**Authors:** Kristina Hmeljak Sangawa

**Affiliations:** 1 University of Ljubljana, Ljubljana, Slovenia

The article describes a system for digitising vernacular reading glosses of the *wokototen* type in classical Chinese texts, presents the results of quantitative measurements of the readings, shapes and positions of such glosses in summaries (compiled by H. Tsukishima) of *wokototen* markings used in actual *kunten* materials, and compares these findings with data from 26 *wokototen* 'comprehensive' charts of different schools compiled by Nakata and Tsukishima.

 Not surprisingly, the results show that simple shapes (dots or lines that can be written with one stroke) are the most common markings, with dots  (' *ten*' in ' *wokototen*' literally means 'dot') being by far the most common; that the most frequent readings are marked by dots, and that most markings appear at the centre or at the four corners overlapping the character they mark. It was also found that markings not overlapping characters are most commonly found on the right of the character, as is still the case for phonetic guides (furigana) in contemporary Japanese.

 Some differences were found between the summaries of *wokototen* glosses in *kunten* materials and the 26 *wokototen* charts, with regard to the most frequent readings and the most frequent position of markings.

 The quantitative findings are not surprising, but do empirically confirm common observations. As the authors note in their conclusion, the most important contribution of this research is the coding and processing method used to digitise *wokototen* markings, described in detail and complete with code that can be used in the future to extend the digitisation and semi-automatic analysis to other material.

 One further important aspect of this article is that it is written in English. The majority of published research on Japanese vernacular readings of classical Chinese texts is written in Japanese, and is therefore not accessible to scholars from other areas of research such as the history of writing, language contact etc., who may not be versed in Japanese. This is also reflected in the list of references of this article, which only includes sources in Japanese (the first three in the list – previous papers by the same authors – are quoted with their English titles, but are actually papers published in Japanese with only an English title and abstract). Given the usefulness of the proposed method for digitisation and processing of text with complex markings and glosses, the fact that it is written in English makes it accessible to a wider audience and thus more likely to be used and built upon.

 The terminology used follows the tradition of Japanese research on this topic and mostly consists of Japanese terms in romanised form. This is somewhat surprising, considering that two of the authors are also co-authors of a thoroughly explained proposal for English terminology to be used in research on vernacular readings of classical Chinese texts (Whitman et al. 2010, http://conf.ling.cornell.edu/whitman/WhitmanAlberizziTsukimotoKosukegawa2010Toward.pdf), where they propose using self-explanatory English translations instead of romanised renditions of Japanese terms, such as "vernacular reading" instead of *kundoku* or "gloss" instead of *kunten*. All terms in this article are, however, clearly explained and thus accessible also to readers who may not be familiar with the Japanese terminology.

 I have a few minor formal suggestions for a possible revised version of the article.

 Japanese long vowels could be romanised more consistently: in the list of references, the long vowel ō is sometimes spelled 'ou' following kana orthography (e.g. 14. Kegonkyou, 15. juyou, 16. Hakubutsukanzou), once 'oh' (Ohtsubo, but misspelled Ohtshbo), and sometimes not marked as long (Kazamashobo, Kegonkyo, Shiryo - misspelled as Shiro). The long vowel ū is not marked as such (kenkyu, ronbunshu etc.).

 The reference list would be more reader-friendly in a more consistent and complete format, e.g. following the Generic style rules for linguistics  [ www.eva.mpg.de/linguistics/past-research-resources/resources/generic-style-rules/]. Specifically, author names would be more easily searchable and less ambiguous if given in both romanized form and Japanese script, and titles more accessible if quoted consistently with both the romanised form and the original Japanese script, followed by an English translation. In the present version of the article, works with English alternative titles (1, 2, 3) are cited only in English, some (6, 8, 10, 11, 12, 15, which originally have no English title) are cited in Japanese script and with an English translation of the title probably provided by the authors, without a romanised transcription of the Japanese title, while other titles (4, 5, 7, 13, 14, 16, 17, 18) are given in romanised form and Japanese script, but without an English translation of the title. For example, the following citation:

 Ohtshbo H: *A study of the kunten language* (訓点語の研究). Kazamashobo; 1961

 would be more informative and accurate in this form:

 Ōtsubo H (大坪併治): *Kuntengo no kenkyū* (訓点語の研究) [A study of the kunten language]. Kazamashobō; 1961

 A few typos should be corrected:

 p. 3 (Target of research) the meaning of a wokototen markings -> marking

 p. 8 (Conclusion) Kunten -> *kunten*

 Kunten Material -> *kunten* material

 (Data availability) can be obtained and search -> searched

 p. 9 (References)

 6. catalogue -> Catalogue

 9. Ohtshbo -> Ōtsubo  /or/  Ootsubo

 12. yukoshoin -> Kyūkoshoin

 18. Shiro -> Shiryou

 Notwithstanding these suggestions for very minor corrections, the article is an important contribution to the study of Japanese vernacular readings of classic Chinese texts, and a welcome introduction of the proposed digitisation method to an international audience.

Is the work clearly and accurately presented and does it cite the current literature?

Yes

If applicable, is the statistical analysis and its interpretation appropriate?

Yes

Are all the source data underlying the results available to ensure full reproducibility?

Yes

Is the study design appropriate and is the work technically sound?

Yes

Are the conclusions drawn adequately supported by the results?

Yes

Are sufficient details of methods and analysis provided to allow replication by others?

Yes

Reviewer Expertise:

History of Japanese writing

I confirm that I have read this submission and believe that I have an appropriate level of expertise to confirm that it is of an acceptable scientific standard.

# Reviewer report: Reviewer response for version 1

**DOI:** 10.5256/f1000research.144061.r174044

**Published:** 2023-08-10

**Authors:** Kiyonari Nagasaki

**Affiliations:** 1 International Institute for Digital Humanities, The University of Tokyo, Tokyo, Japan

I acknowledge the potential significance of the research presented in this paper, which offers an insightful foundation for discussing matters related to pre-modern international exchange and translation. The findings are compelling, contingent upon further corroboration through subsequent research.

 However, having perused the manuscript in detail, I wish to raise two points for your kind consideration and, if possible, rectification.

 Firstly, our attention was drawn to the sequence of "te", "wo", and "ni". As per the frequency order established in your study, they should be listed as "te", "wo", and "ni". However, we have observed instances where the sequence appears as "te", "ni", and "wo". Unless there is a specific reason for this inconsistency, we recommend standardizing the order for improved coherence.

 Secondly, I noticed that the analysis carried out in this study could arguably be conducted without the use of digital technology. In order to enhance the position of your paper within the landscape of existing research and amplify its overall value, it would be beneficial to offer a clearer context. Specifically, it would be helpful if you could clarify whether no such analysis existed prior to the advent of digital technology, or if there was any similar analysis previously. If the latter is not the case, kindly offer a rationale for the absence of such analyses in the past. This contextual detail will further enrich the relevance of your work within the wider field of study.

 I request your thoughtful consideration of these points.

Is the work clearly and accurately presented and does it cite the current literature?

Yes

If applicable, is the statistical analysis and its interpretation appropriate?

Yes

Are all the source data underlying the results available to ensure full reproducibility?

Yes

Is the study design appropriate and is the work technically sound?

Yes

Are the conclusions drawn adequately supported by the results?

Yes

Are sufficient details of methods and analysis provided to allow replication by others?

Yes

Reviewer Expertise:

Digital Humanities and Buddhist studies

I confirm that I have read this submission and believe that I have an appropriate level of expertise to confirm that it is of an acceptable scientific standard.

# Reviewer report: Reviewer response for version 1

**DOI:** 10.5256/f1000research.144061.r186365

**Published:** 2023-08-10

**Authors:** Pádraic Moran

**Affiliations:** 1 Department of Classics (School of Languages, Literatures and Cultures), University of Galway, Galway, County Galway, Ireland

This article is a valuable study of *wokotoken *annotations, part of the Japanese *kunten *system of annotating Chinese/Sinitic texts. Its value lies in its computational approach. By counting the large number of complex *wokotoken *systems collected in Tsukishima 1986, the authors demonstrate very clear distribution patterns, which present new insights into Japanese annotation practices. The information is clearly and carefully presented, and the implications for future research (presented the Conclusion section) are very significant. This article will be of interest to specialists in *kunten* *. *It should also interest researchers on glossing more generally, since the computational approach adopted here could be a model for specialists in other traditions to follow.

 1) I saw just one significant error:

 p. 5: ‘Examples are shown in the 'カ/ナ' in the top left-hand corner (-2, -2) of the fifth tsubo in Figure 1.’ This appears to be the third tsubo (middle left), not the fifth. (Also ‘カ/ナ’in the text is confusing, since the figure has the reverse: ナ/カ. Recommend to change the text accordingly.)

 2) I have two recommendations:

 p. 5, table 1: Several shapes are followed by a sign ↑or ↓. These seem to require explanation.

 p. 7, figure 2: Although the figure is extremely helpful, it is a little hard to read. The Y-axis runs from +3 on top to -3 on the bottom. However, the corresponding table 5 has -3 on top and +3 on the bottom. This is more intuitive, since -3 represents the top of a character. Recommend to reverse the Y-axis in figure 2.

 3) The following minor errors should be corrected:

 p. 3:

 ‘around from the Heian period…’—sense of ‘around from’ is not clear; recommend to delete ‘around’

 ‘charts are used as means’

 ‘ *wokototen *is mostly found’ > ‘ *wokototen* are mostly found’

 p. 4:

 ‘replacing it with the similar character in Unicode’—‘the’ > ‘a’

 ‘The 124 characters… is shown’—‘is’ > ‘are’

 p. 5:

 ‘This digitization method… are described in’—‘are’ > ‘is’

 ‘These methods of the Korean studies’—‘These’ > ‘The’

 ‘… has the same concept’—‘has’ > ‘have’

 ‘The programme was written in C#’—‘The’ > ‘A’

 ‘Sharp’ > ‘Shape’

 ‘Examples are shown’ > ‘An example is shown’

 ‘into two separate totals. The one of *wokototen *of the shape ‘L’ reading ‘カ’ and the other of *wokototen *of the shape 'L' reading 'ナ.’ > ‘into two separate items, one for the *wokototen *shape ‘L’ reading ‘カ’ and the other for the *wokototen *shape 'L' reading 'ナ’.’

 p. 7:

 ‘six *wokototen *chart’—‘chart’ > ‘charts’

 p. 8:

 ‘who compiled a chart’ > ‘who compiled charts’

Is the work clearly and accurately presented and does it cite the current literature?

Yes

If applicable, is the statistical analysis and its interpretation appropriate?

Yes

Are all the source data underlying the results available to ensure full reproducibility?

Yes

Is the study design appropriate and is the work technically sound?

Yes

Are the conclusions drawn adequately supported by the results?

Yes

Are sufficient details of methods and analysis provided to allow replication by others?

Yes

Reviewer Expertise:

Comparative glossing (annotation) practices generally, specialising in the European Latin tradition, but with an interest in Japanese glossing. Digital Humanities and computational analysis of texts.

I confirm that I have read this submission and believe that I have an appropriate level of expertise to confirm that it is of an acceptable scientific standard.

# Reviewer report: Reviewer response for version 1

**DOI:** 10.5256/f1000research.144061.r186344

**Published:** 2023-07-17

**Authors:** Brian Steininger

**Affiliations:** 1 Department of East Asian Studies, Princeton University, Princeton, New Jersey, USA

The article presents a format for digitizing data on vernacular reading glosses in the okototen format, applied to classical Chinese texts in premodern Japan. Using this methodology, the authors compare data from "comprehensive charts" (prescriptive instructions for producing glosses kept by a particular school) and "inductive charts" (diagrams of actual glosses found in primary sources made by contemporary researchers--in this case, the work of Tsukishima Hiroshi). This is an important distinction. The statistical results of the survey contain few major surprises, though there is a somewhat interesting discrepancy in regard to the use of the space to the right of the character. As indicated in the conclusion, the major importance of this research is as foundational work building towards essential new tools for digitization and semi-automated analysis of kunten materials.

Is the work clearly and accurately presented and does it cite the current literature?

Yes

If applicable, is the statistical analysis and its interpretation appropriate?

Yes

Are all the source data underlying the results available to ensure full reproducibility?

Yes

Is the study design appropriate and is the work technically sound?

Yes

Are the conclusions drawn adequately supported by the results?

Yes

Are sufficient details of methods and analysis provided to allow replication by others?

Yes

Reviewer Expertise:

Reception of Chinese literature in early and medieval Japan

I confirm that I have read this submission and believe that I have an appropriate level of expertise to confirm that it is of an acceptable scientific standard.
